# The Key Role of 3D TEE in Assessing the Morphology of Degenerative Mitral Valve Regurgitation

**DOI:** 10.3390/jcdd11110342

**Published:** 2024-10-28

**Authors:** Francesco Fulvio Faletra, Eluisa La Franca, Laura Anna Leo, Leyla Elif Sade, William Katz, Francesco Musumeci, Caterina Gandolfo, Michele Pilato, Manlio Cipriani

**Affiliations:** 1 IRCCS-ISMETT (Istituto Mediterraneo per i Trapianti e Terapie ad Alta Specializzazione), UPMCI (University of Pittsburgh Medical Center Italy), 90100 Palermo, Italy; elafranca@ismett.edu (E.L.F.); fmusumeci@ismett.edu (F.M.); cgandolfo@ismett.edu (C.G.); mpilato@ismett.edu (M.P.); mcipriani@ismett.edu (M.C.); 2 Istituto Cardiocentro Ticino, 6900 Lugano, Switzerland; lauraanna.leo@eoc.ch; 3 Heart and Vascular Institute, University of Pittsburgh Medical Center, School of Medicine, University of Pittsburgh, Pittsburgh, PA 15213, USA; sadele2@upmc.edu (L.E.S.); katzwe@upmc.edu (W.K.)

**Keywords:** 3D echocardiography, mitral valve apparatus, mitral valve regurgitation

## Abstract

Two-dimensional transthoracic echocardiography (2D TTE) and two-dimensional transesophageal echocardiography (2D TEE) are regarded as the main imaging techniques for the assessment of degenerative mitral valve regurgitation (DMVR). However, describing the complex morphology of DMVR with 2D TTE and 2D TEE remains at the very least challenging. Three-dimensional (3D) TEE is an ideal technique for illustrating the extremely variable morphology of DMVR, providing images of unparalleled quality in terms of anatomical detail. In this review, we describe the key role of 3D TEE in various morphological scenarios that reflect everyday experiences in an echocardiographic laboratory. We also discuss the growing role of 3D TEE in mitral valve (MV) transcatheter edge-to-edge repair (TEER) and new modalities such as photorealistic and transparent displays, surface rendering parametric color maps, new algorithms for MVR quantification, and the potential role of new mini-TEE probes in adult patients with DMVR.

## 1. Introduction

Two-dimensional transthoracic echocardiography (2D TTE) is widely regarded as the first-line imaging technique for diagnosing degenerative mitral valve regurgitation (DMVR), providing essential data on lesion type, severity of regurgitation, left and right ventricular function, systolic pulmonary pressure, and the presence of other associated valve lesions [1].

Two-dimensional transesophageal echocardiography (2D TEE) is recommended as a second-line approach when 2D TTE provides insufficient or discordant data for a complete diagnosis [2]. Currently, 2D TEE is extensively used by cardiologists and echocardiographers in the echo lab, cardio-anesthesiologists in the operating room, and cardiologists/imagers in the hybrid room during transcatheter mitral valve (MV) edge-to-edge repair (TEER) [2,3].

The popularity of 2D TEE among cardiologists is based on several factors. Compared to 2D TTE, 2D TEE benefits from (a) negligible distortion of the ultrasound beam passing through the relatively thin esophageal wall, (b) close proximity of the intra-esophageal probe to the posterior structures of the heart, (c) the use of high-frequency transducers, and d) a potentially endless number of cut planes. The transducer can, in fact, be advanced and withdrawn through the esophagus and the stomach, and there are five standardized levels (upper, mid, and deep esophageal, as well as transgastric and deep transgastric). For each level, the transducer angle can be electronically rotated forward and backward from 0° to 180° degrees. Using the two control wheels in the probe handle, the transducer can be anteflexed, retroflexed, or flexed to the right and left, generating many longitudinal, transverse, and oblique tomographic planes. Finally, it can be turned 360° clockwise and anticlockwise on its axis for each level.

Despite its capabilities and universal use, 2D TEE has several limitations in assessing the extremely variable morphology of DMVR. First, 2D TEE provides only thin tomographic slices, where leaflets appear as two subtle white lines moving up and down against a black background. Second, the position of the heart (horizontal, oblique, vertical) and the position of the esophagus (middle, rightwards, or leftwards to the posterior left atrial wall) change from patient to patient. Thus, rigid coordinates for visualizing a given prolapsing segment may be valid for one patient but not for another. Thus, in the end, the number of useful and diagnostic cross-sections is limited. Third, though experienced echocardiographers may instinctively reconstruct in their mind a 3D image derived from multiple 2D sections, this may not be entirely true for less experienced echocardiographers.

**Figure 1:**
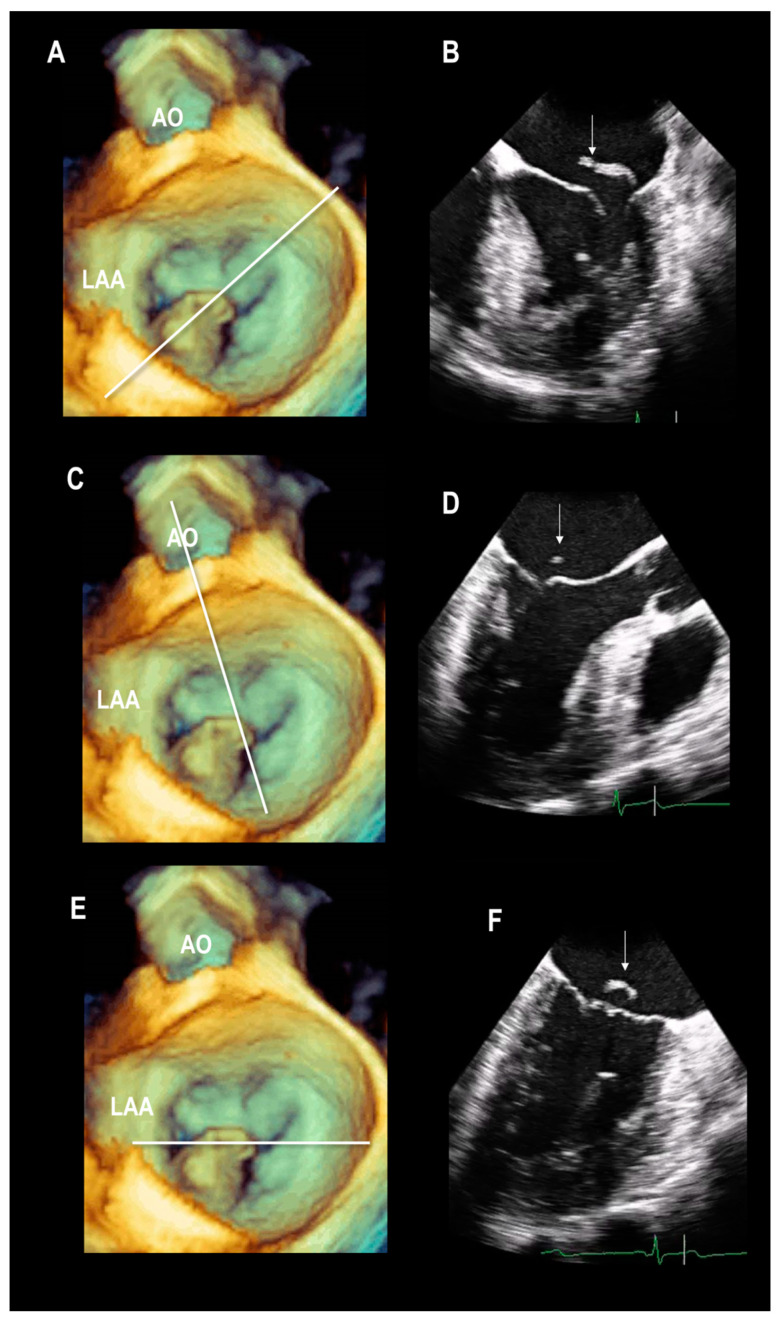
(**A**,**C**,**E**) 3D TEE images showing a flail scallop located in the central-lateral segment of the posterior leaflets. (**A**) The line corresponds to the 2D TEE four-chamber view shown in panel (**B**). This cross-section visualizes the flail segment from its hinge line to the free margin (arrow). (**C**) In this panel, the line corresponds to the 2D TEE long-axis view shown in panel (**D**). In this cross-section, only a small part of the free edge of the flail leaflet is visualized (arrow). (**E**) In this panel, the cut plane that transects the valve near the two commissures corresponds to the 2D TEE bi-commissural view shown in panel (**F**). Only a part of the flail leaflets is visualized (arrow).

In Figure 1, 3D TEE images in panels A, C, and E show a flail in the posterior leaflet’s central-lateral segment. The lines correspond to the cut planes illustrated in panels B, D, and F. Admittedly, looking only at the 2D TEE panels, a precise 3D mental reconstruction of this simple DMVR morphology might be difficult.

Therefore, describing the complex morphology of DMVR with 2D TEE remains at the very least challenging. It is not by chance that cardiologists often focus their attention on the severity of MR and the timing of surgical intervention when using 2D TEE, while a detailed morphological description is usually lacking and limited to the localization of the affected segment and mechanisms of regurgitation.

### Three-Dimensional Echocardiography

The current generations of matrix array transducers have benefited from a series of remarkable technological advancements over just a few years. New, more efficient piezoelectric crystals, improved crystal manufacturing processes (making it possible to obtain crystals as small as 200 microns), miniaturization of electronic circuitries (making it possible to fit ∼2500 crystals into the tip of a normal-sized TEE probe), and advances in computer technology (allowing thousands of elements to be active simultaneously) have made 3D TEE what we know today [4]. Table 1 summarizes these technological developments.

**Figure 2:**
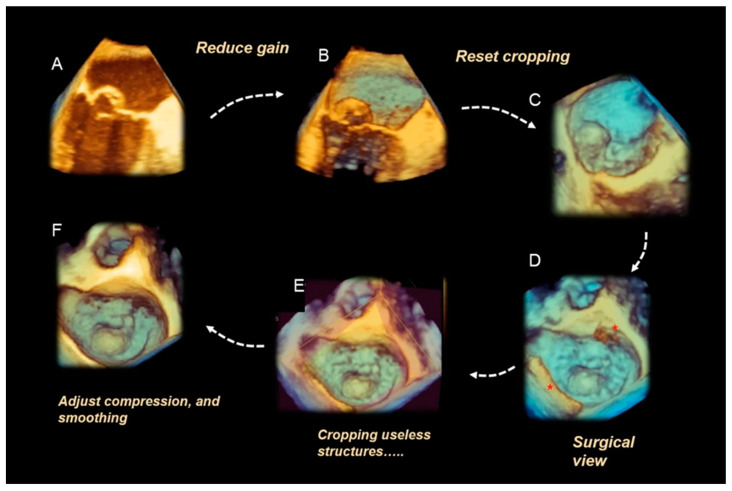
(**A**) Starting acquisition. Although an acquisition with a little over-gain is recommended, too much gain reduces the perception of the third dimension. Thus, adjusting the gain is the first step to increasing the perception of the third dimension, visualizing structures covered by static noise. (**B**) Often, the system automatically crops the pyramidal data set to show structures inside it, so the next step is to reset the cropping. (**C**) This is an oblique view. Although this particular perspective may be useful, it is convenient first to orientate the volumetric data set to obtain the classic surgical view. (**D**) In this view, the red asterisks mark static noise and a part of the atrial wall. Both are useless and should be cropped (**E**). (**F**) The final step is to slightly reduce the compression (to enhance the margins) and slightly increase the smoothing to make the leaflet surface smoother.

From the beginning, it was clear that 3D TEE was an ideal technique for illustrating the extremely variable morphology of DMVR, providing images with unparalleled anatomical detail [5]. The dynamic nature of DMVR requires an acquisition modality capable of high spatial resolution (to identify the fine morphological details of the valve) and high temporal resolution (to allow an accurate frame-by-frame analysis without large temporal gaps between frames). Zoom ECG-gated multi-beat acquisition or single-beat zoom modality acquisition are the best acquisition modalities for visualizing DMVR [4]. Properly tuning the gain settings, cropping useless structures, and adjusting compression and smoothing may further improve the image quality, making small anatomic details visible. Figure 2 shows an example of step-by-step adjustments to the 3D data set to obtain the best possible images in a patient with a P2 flail.

We believe that the large variability in DMVR morphology can be accurately described by 3D TEE [5]. This review aims to summarize the advantages of 3D TEE in assessing DMVR by describing various morphological scenarios that reflect everyday experiences in an echocardiographic laboratory. A secondary aim is to evaluate the impact on DMVR assessment of new technologies such as photorealistic and transparent displays, surface rendering parametric color maps, new algorithms for MVR quantification, and the new mini-TEE probe.

## 2. Morphological Scenarios

### 2.1. Scenario # 1

#### Multiple Perspectives

**Figure 3:**
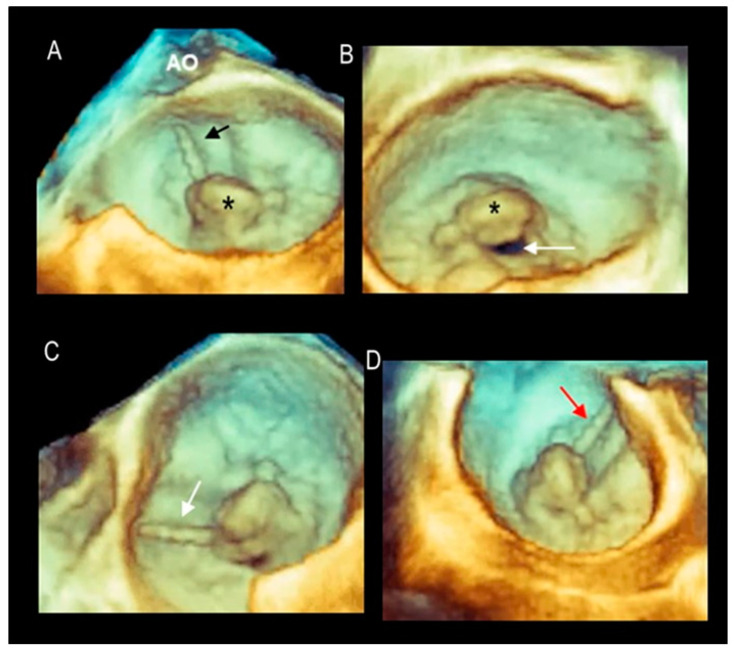
(**A**) Surgical view. The arrow in the image points to the ruptured chordae tendineae, and the asterisk marks the prolapse of the mid-scallop. The aorta (AO) is seen at ~12 o’clock. (**B**) Anterior view. This view is obtained by rotating the 3D data set 180° and angulating down-up. The view allows visualization of the anatomic regurgitating orifice (arrow). (**C**,**D**) Left-to-right and right-to-left tangential views. These views allow a better perception of the protrusion of the prolapsing tissue into the left atrium and at the same time visualization of the commissural areas. The arrows point to the ruptured chordae tendineae.

One of the extraordinary abilities of 3D TEE is the fact that, once acquired, the pyramidal data set can be angulated and rotated in any direction, truly providing countless perspectives. The non-surgical views, and in particular the so-called “angled or tangential views” first described by Biaggi et al. [6], provide additional data compared to the surgical view alone. Figure 3 shows the most useful angled perspectives.

### 2.2. Scenario # 2

#### Phenotypes of DMVR

The term DMVR includes a wide spectrum of diseases ranging from prolapse/flail of an isolated segment in an otherwise normal-sized valve (fibroelastic deficiency [FED]) to multi-segment prolapse/flail (Barlow’s disease). Intermediate stages of the disease, namely FED+ and forme fruste, have been described by Adams et al. [7] in an attempt to provide a more systematic categorization of the continuous spectrum of DMVR. Three-dimensional TEE has proven to be an ideal imaging tool for visualizing these phenotypes. Table 2 shows a brief description of these phenotypes and the corresponding 3D TEE images.

### 2.3. Scenario # 3

#### Cleft and Cleft-like Indentations

In a normal mitral valve, the anterior leaflet (AML) does not present scallops, while the posterior mitral leaflet (PML) is usually divided into three scallops by two indentations. These indentations typically do not extend more than half the depth of the two adjacent scallops. Carpentier used these indentations to facilitate segmental analysis of the valve [8]. Functionally, they allow the scallops to squeeze together, effectively sealing the curved closure apposition line and facilitating the full opening of the posterior leaflet, otherwise impeded by its curved insertion on the posterior annulus. In the literature, the term cleft has been used to describe a gap that divides the AML into two halves as part of the atrioventricular (AV) septal defect [9]. From an anatomical point of view, this cleft is a left commissure between the two bridging leaflets of the common five-leaflet atrioventricular valve. An isolated AML cleft not associated with the AV septal defect is rather uncommon [10], and it is unclear whether or not it represents a kind of form fruste of the more complex AV defect.

A cleft of the anterior mitral leaflet has never been described in DMVR, and a cleft separating the mid scallop (P2) of the posterior leaflet into two halves is rarely seen [11]. Conversely, cleft-like indentations occupying the position of normal indentations are by far more frequent. Unlike normal indentations, these cleft-like indentations extend more than half the depth of the two adjacent scallops, not infrequently reaching the hinge line. They are always found around prolapse or flail segments. Multi-scallop prolapse can have multiple cleft-like indentations. They usually cause regurgitation, likely due to a combination of the following mechanisms: (a) enlargement of the mitral annulus stretching the scallops and increasing the gap between them, (b) excessive leaflet protrusion with a persistent gap between the prolapsing and non-prolapsing segments, and (c) insufficient leaflet tissue to cover the scallop separation [11].

Cleft-like indentations without regurgitation must be regarded with suspicion. Indeed, they may become a cause for early failure after MV TEER. The repaired valve may be distorted in some way, enlarging the gap between scallops and causing unexpected MR.

**Figure 4:**
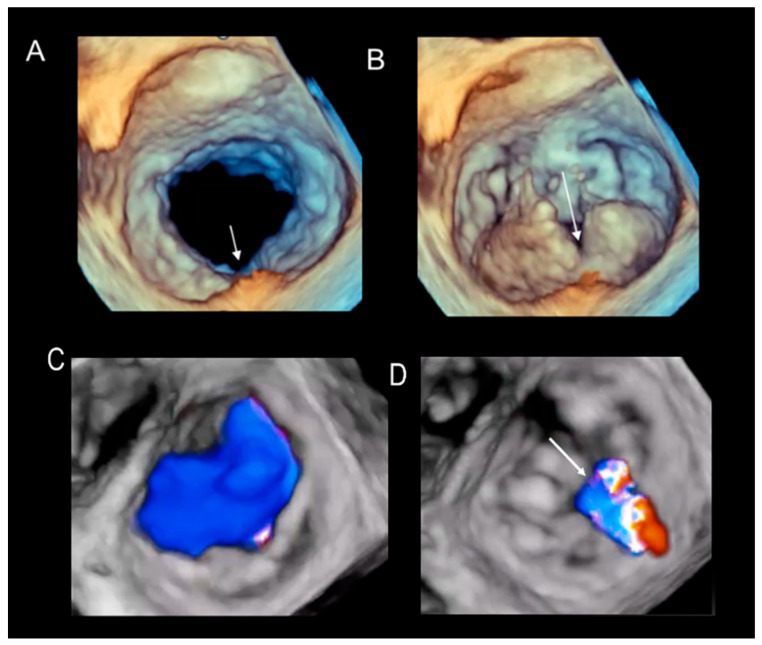
(**A**,**B**) Deep cleft (arrow) separating a large P2 into two halves, displayed in diastole (**A**) and systole (**B**). (**C**,**D**) The same patient using 3D TEE color Doppler. The images show that, in systole (**D**), the regurgitant jet flows across the cleft.

**Figure 5:**
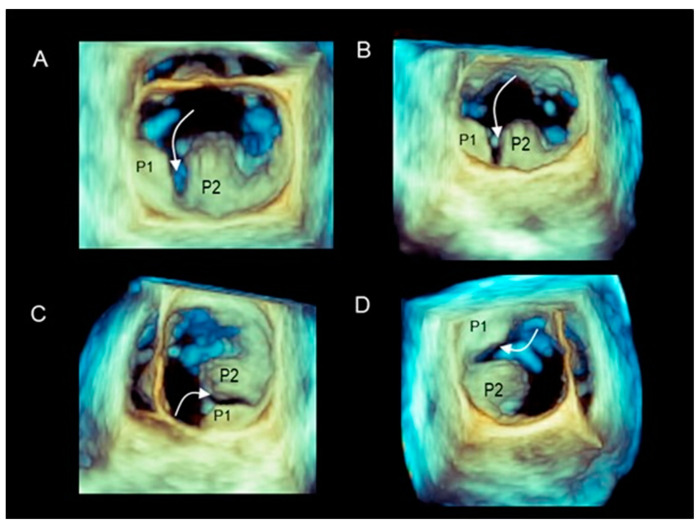
(**A**–**D**) Large cleft-like indentation (curved arrow) positioned between P1 and P2, seen from different perspectives.

Two-dimensional TEE cannot depict the precise anatomic characteristics of the cleft-like indentations. Conversely, 3D TEE appears to be particularly suitable for precisely depicting their length and shape from both atrial and ventricular perspectives or angled views. Figure 4 and Figure 5 show 3D TEE examples of clefts and cleft-like indentations.

### 2.4. Scenario # 4

#### Different Shapes of P2 Prolapse, Partial Prolapse, and Commissural Prolapse

**Figure 6:**
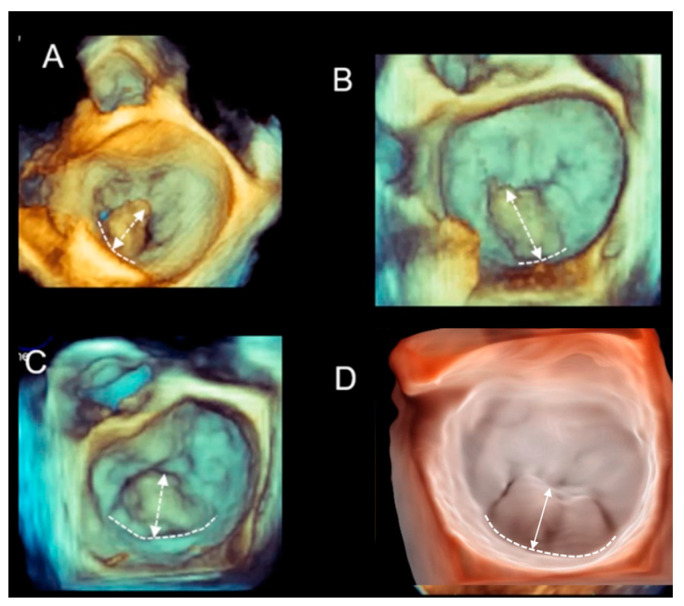
(**A**–**D**) Different morphologies of P2 prolapse. Each of them may require a different valve repair strategy. The curved dotted line marks the width of the prolapsing tissue at its hinge line. The double-headed arrow marks the distance between the free margin and the hinge line of the prolapsing tissue. The image in panel (**D**) has been acquired using a photorealistic view (see below).

Surgical inspection and anatomic specimens have shown that no two P2 prolapses/flails are identical, and this has an impact on the choice of valve repair techniques; narrow-based prolapse may require only a simple triangular resection completed by annuloplasty, while broad-based P2 prolapse may require quadrangular resection, sliding technique, and annuloplasty. A very broad-based P2 prolapse/flail may require neo-chordae implantation [12]. Tomographic cross-sections of 2D TEE often fail to define with certainty the anatomical characteristics of an individual P2 prolapse. Conversely, 3D TEE appears to be the ideal technique to accurately define the site, size, and shape of the prolapsing tissue. Moreover, 3D TEE allows the easy distinction between an ideal (i.e., single scallop prolapse/flail, tissue exuberance, absence of annular calcification, and moderate annular dilation) and a challenging pathoanatomy (i.e., FED, Barlow’s disease, flail of the anterior leaflet, deep cleft, and extensive calcification), and this is extremely important for planning surgical or percutaneous repair [13]. Figure 6 shows different morphologies of P2 prolapse.

**Figure 7:**
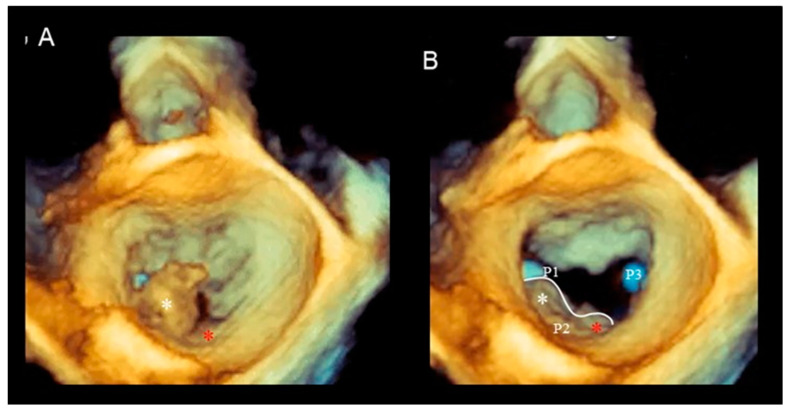
(**A**) Systolic frame. P2 flail (white asterisk). (**B**) In diastole, it can be observed that the P2 scallop is large, and the prolapsing segment occupies the lateral part of P2, while the medial part does not prolapse (red asterisk). The white line marks the free margin of P2. Please note as the P1 and P3 scallops are smaller than P2.

In a large P2 prolapse, frequently only one part of the scallop will prolapse or flail, while the remaining part will not. Figure 7 shows one example of this partial prolapse.

**Figure 8:**
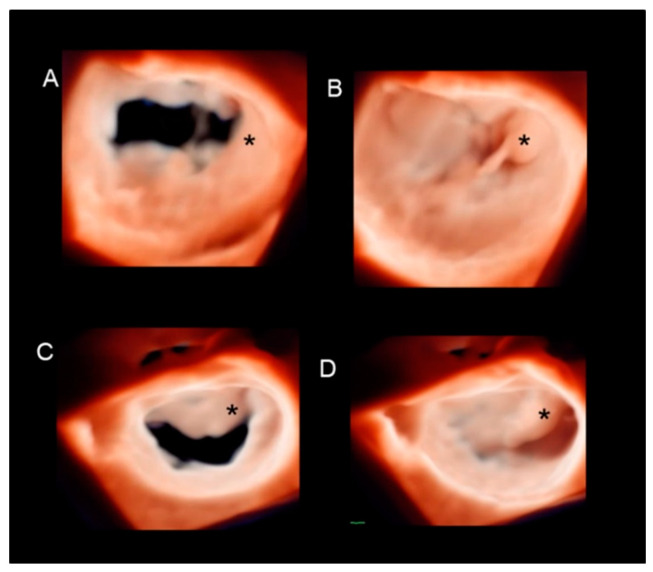
(**A**,**B**) Commissural prolapse originating from a P3 scallop with ruptured chordae tendineae. (**C**,**D**) Commissural prolapse originating from the A3 segment. The asterisks mark the area of prolapsing tissue. The images have been acquired using a photorealistic view (see below).

Commissural prolapse can be found either in the context of Barlow’s disease or as an isolated lesion. The morphology of commissural prolapse is extremely variable. Commissural prolapse may originate from a supplementary commissural scallop or, more frequently, from prolapse/flail of commissural regions. Three-dimensional TEE remains the best imaging modality for visualizing the site, origin, shape, and size of commissural prolapse, aiding in planning the most appropriate surgical or percutaneous technique [14]. Figure 8 shows 3D TEE examples of commissural prolapse.

### 2.5. Scenario # 5

#### Secondary Lesions

**Figure 9:**
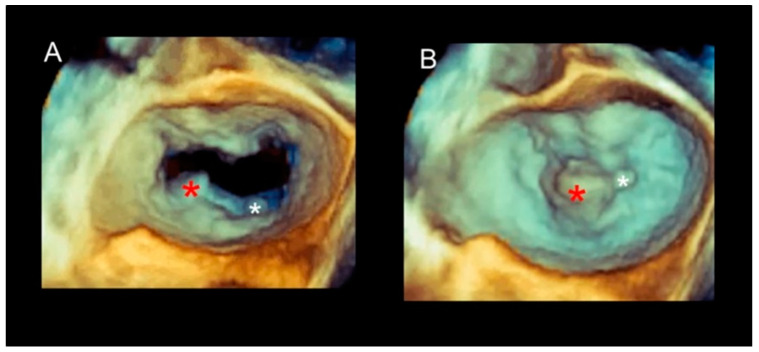
P2 flail with ruptured chordae tendineae in diastole (**A**) and in systole (**B**). The red asterisk marks the flail of the lateral part of P2, while the white asterisk points at a small prolapse of the medial part of P2. The latter could not be recognized in the operating room when the heart was still and flaccid.

Surgical inspection is indisputably the gold standard. Several studies have shown that 3D TEE, when compared with surgical inspection, is highly accurate in defining the multiform aspects of DMVR [14,15]. However, in 2011, La Canna et al. demonstrated that while dominant lesions (i.e., excursion of the prolapsing leaflet above the annulus > 5 mm) were always confirmed at surgery, a significant percentage of minor lesions seen on 3D TEE was not recognized at the moment of surgical inspection, despite skillful observation by surgeons with great experience in MV repair [16]. The authors speculated that one of the reasons for this discrepancy was the fact that valve morphology was assessed during an arrested, empty heart and, therefore, small lesions diagnosed with 3D TEE in awake patients might not have been recognized. Therefore, while surgical inspection remains the gold standard for dominant lesions, the non-beating, flaccid heart might preclude the detection of smaller lesions. Discovering small lesions may be crucial and should require full consideration by surgeons to obtain durable MV repair. Indeed, in long-term follow-up, patients undergoing MV repair of only the dominant prolapse may require reoperation for the recurrence of severe mitral regurgitation due to the progression of untreated small prolapses [17]. Thus, 3D TEE could be considered the reference standard for small, dynamic lesions. Figure 9 shows one of these small lesions.

### 2.6. Scenario # 6

#### Mitral Annular Calcification

Mitral annular (MA) calcification is common in patients with DMVD. Extensive MA calcification profoundly alters the dynamic of the annulus, often worsening regurgitation. Indeed, calcifications may extend to the leaflets, making them stiff and less pliable, or may accumulate on the ventricular and atrial sides of the leaflet hinge line, reducing physiological sphincteric contraction and making surgical repair much more challenging [18]. Typically, calcification affects the posterior MA more frequently and more extensively. The posterior annulus is constituted by a discontinuous dense collagenous tissue. In the gap between the fibrous tissues, the posterior leaflet is inserted directly into the muscular tissue. It may be speculated that this anatomical arrangement, while facilitating annular contraction, might cause microinjuries at the leaflet hinge line during contraction and relaxation, initiating the deposition of microcalcifications which, over decades, may coalesce into the rigid band that becomes macroscopically visible [19].

**Figure 10:**
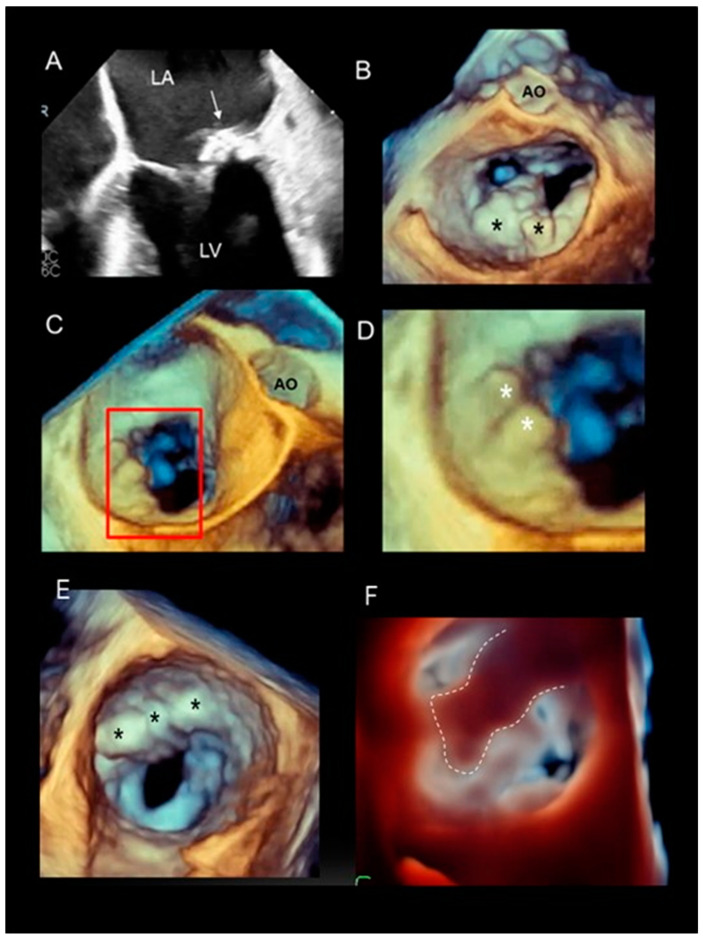
(**A**) Two-dimensional TEE showing extensive calcification of the posterior annulus (arrow). Calcifications are distinguishable because they are brighter in comparison with the surrounding structures. (**B**) The same calcification is visualized with 3D TEE. Calcifications (asterisks) are depicted with the same shades of color as the surrounding structures. (**C**) Three-dimensional TEE of annular calcification seen in oblique perspective. The red square includes the structures that are magnified in panel (**D**). Calcifications appear to be bumping and protruding into the left atrium (asterisks). This phenomenon is artefactual and is due to the blooming effect. (**E**) Three-dimensional TEE showing calcifications protruding into the left atrium (asterisks). (**F**) Photorealistic effect. When the artificial light is placed beyond the calcifications, they appear darker than surrounding structures and their borders can be better defined (dotted line). See below.

Using 2D TEE, annular calcification appears as an echo-dense, shelf-like bright structure with an irregular, lumpy appearance (Figure 10A). Unfortunately, calcification is one of the “Achilles’ heels” of 3D TEE. The mix of blue-bronze-beige-yellow used in 3D TEE has been designed to increase depth perception. As a consequence, calcified and non-calcified tissues lying at the same depth are shown with the same shades of color, making them practically indistinguishable from one another (Figure 10B). Some tips and tricks may help to distinguish calcium from other surrounding structures: first, unlike non-calcified structures, calcium does not usually move during the cardiac cycle; and second, calcified structures suffer from blooming artifacts, usually appearing larger than they actually are and artifactually protruding into the left atrium (Figure 10C,D). The photorealistic display (see below) enables the placement of a mobile light source beyond the valve. Since the calcified areas impede the artificial light passing through them, the images of the calcifications appear darker than surrounding structures making them easier to identify (Figure 10E,F).

### 2.7. Scenario # 7

#### 3D TEE During MV Transcatheter Edge-to-Edge in DMVR

Early experience with the use of MV transcatheter edge-to-edge repair (TEER) in DMVR was confined to patients with favorable anatomy, i.e., single clip on A2-P2 defect, valve area > 4 cm^2^, flail gap < 10 mm and flail width < 15 mm [20]. Over the years, with increasing operator experience and new, more performant catheters and devices, TEER has expanded to different morphologies, and currently, there are only a few morphological contraindications [21]. One is a mitral valve area < 3 cm^2^, which is best quantified by planimetry using the 3D TEE multiplanar reconstruction modality.

Since the main function of TEER is to capture the mitral leaflets (which are invisible to fluoroscopy), 2D TEE is the primary imaging modality, and fluoroscopy is used as an ancillary technique. Two-dimensional TEE can cover all the steps of MV TEER and is the only available modality for capturing the leaflets. In the last few years, the matrix array transducer has played its role, providing two simultaneous cross-sectional plane views of the same heartbeat, orthogonal to each other. This acquisition modality is currently widely used for many steps of the procedure [21].

Nevertheless, exhaustive reviews have suggested the use of 3D TEE at least in some steps of MV TEER [22,23]. Indeed, 3D TEE has undisputable advantages over 2D TEE: first, the pyramidal data set, encompassing volumetric information, allows the visualization of a long segment of the guide wire and the guide catheter (in a single image); second, 3D TEE displays realistic images of the mitral clip delivery system; third, 3D TEE displays the precise anatomical relationship between the device and the leaflet, especially in positioning the arms of the device perpendicular to the line of coaptation; and fourth, 3D TEE offers a panoramic view of the catheters, system delivery, leaflets, interatrial septum, and atrial wall in a single shot with an acceptable volume rate (~15 volumes per sec). Use of 3D TEE during MV TEER depends on the ability of the imager to provide understandable 3D images rapidly and the interventionalist’s confidence in interpreting these images. Table 3 summarizes the steps in the procedure where 3D TEE is considered superior to 2D TEE.

## 3. Pitfalls and Limitations of 3D TEE

3D TEE suffers from artifacts and pitfalls. One of the major artifacts of 3D TEE that may occur when acquiring images of DMR is the so-called “sticking artifacts”. As previously mentioned, the best imaging modality for acquiring a 3D data set of DMR is the ZOOM multi-beat ECG-gating acquisition. This modality captures sequential narrow sectors (up to 6) and, by arranging them together, produces a large volumetric data set with the highest spatial and temporal resolution. However, a correct acquisition requires regular heart rhythm and breath-holding. Stitching artifacts are defined as an incorrect juxtaposition at the interface between sectors. These artifacts happen any time the acquisition is made during arrhythmias, deep respiration, different lengths of the cardiac cycle, or any transducer or patient movement. In these settings, the positions in the space of the different sectors vary creating these artifacts. Other 3D artifacts such as drop out, shadowing, blurring, and blooming artifacts do not occur or are less relevant when acquiring 3D images of DMR.

## 4. New Technologies

### 4.1. Photorealistic Vision and Transparency

**Figure 11:**
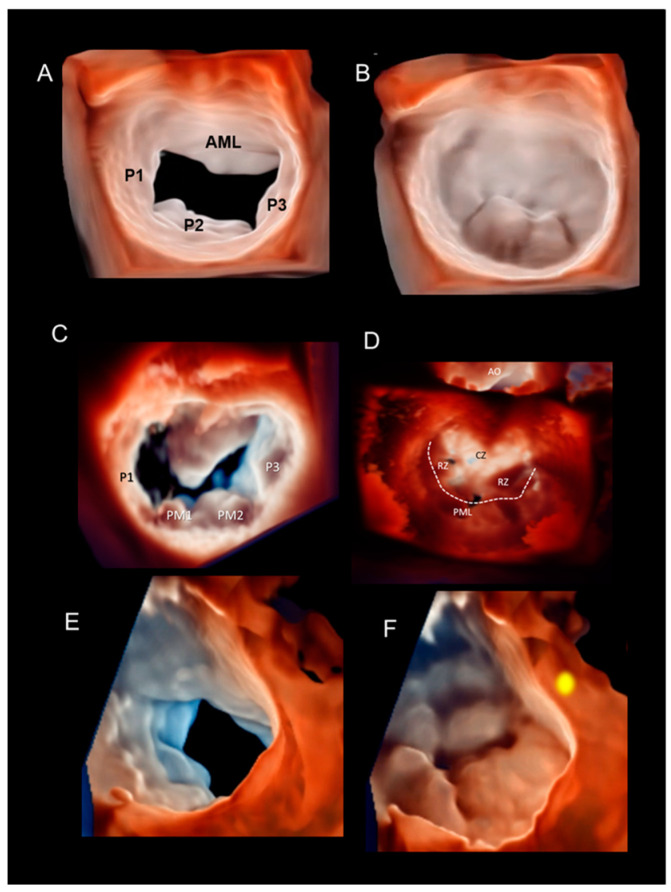
(**A**,**B**) Prolapse of P2 in diastole (panel **A**) and in systole (panel **B**). True view allows perfect definition of the protrusion of the P2 scallop, creating a thin shadow around the prolapsing tissue. (**C**,**D**) Barlow’s disease in diastole (panel **C**) and in systole (panel **D**); the dashed line points at the coaptation line. The light source is beyond the annular plane, creating a transillumination effect. In systole, the clear zone (CZ) of the AML, being thinner than the rough zone (RZ), appears brighter. (**E**,**F**) Barlow’s disease in diastole (panel **E**) and in systole (panel **F**). A clear perception of the protrusion of the entire valve above the annulus is obtained through a combination of tangential view, light source positioned adjacent and lateral to the leaflets (yellow spot), and transparency. AML = anterior mitral leaflet.

A new tool, called photorealistic vision or true view, which uses a mobile, virtual light source has been introduced recently. This algorithm simulates light–tissue interactions, including absorption, scattering, and reflection [24]. The light can be placed anywhere within the volumetric data set, enhancing the digital shadowing of the prolapse/flail segments (Figure 11A,B). There is no preferred location for the light source due to the wide variation in morphology of DMVR. As a general rule, the more shadowing created, the more depth perception is achieved. Particularly interesting is the effect of retro-illumination created when the light is placed beyond the valve (Figure 11C,D), which in DMVR helps to distinguish the clear zone from the rough zone of the AML. Indeed, the clear zone without chordae tendineae insertions is thinner and allows more light to pass through. In the rough zone, the presence of chordae insertions makes this part of the AML thicker, and it appears darker when retro-illuminated. Moreover, as described in Scenario # 6, retro-illumination helps in defining the extension of the calcifications. Another recent tool displays 3D images with varying degrees of transparency of the cardiac structures (glass effect). In general, the variable perception of protrusion of flail segments in DMVR depends on a smart combination of an angled view with a light source tangential to the prolapsing tissue and a certain degree of transparency (Figure 11E,F)

**Figure 12:**
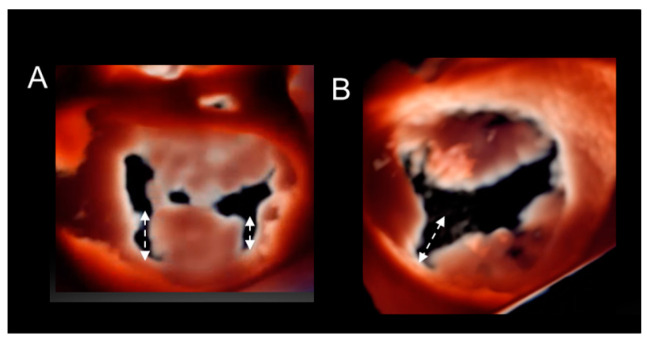
(**A**,**B**) Photorealistic images with the source of light beyond the valve. Large cleft-like indentations (double dotted arrows) are seen from ventricular perspectives.

This retro-illumination is an excellent tool for visualizing cleft-like indentations and appreciating their extension (Figure 12).

### 4.2. Surface Rendering

**Figure 13:**
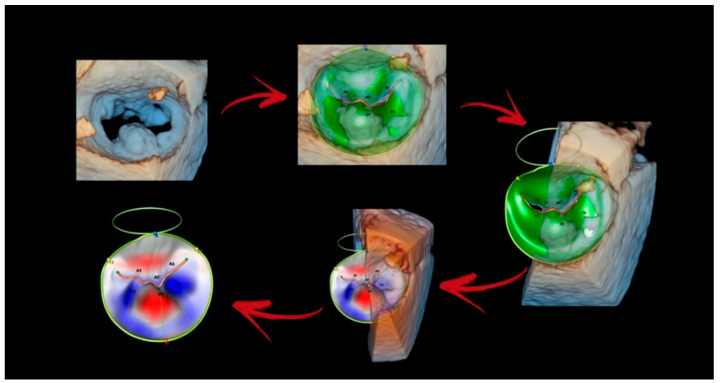
An example of surface rendering using new software that allows the measurement of numerous quantitative parameters in a very short time (see text).

Generally, the 3D morphological features of DMVR are described qualitatively. However, even experienced observers will likely disagree as to which segment is truly prolapsing, especially in multi-scalloped prolapses. Dedicated quantitative software can digitally reconstruct a precise three-dimensional parametric map of the entire valve. This modality depicts the surfaces of structures that have been identified by manual or (semi)automated border tracing utilizing pattern recognition and other forms of AI to identify fiducial anatomic landmarks [4]. The parametric color map overcomes the limitations of qualitative interpretation/description and improves accuracy and reliability while reducing interobserver variability. The new software can automatically quantify annulus dimensions, shape, perimeter, area, height, inter-commissural and anteroposterior diameters, leaflet length/area, prolapse volume/height, and tenting volume/height in a very short time (Figure 13).

## 5. Assessment of Regurgitation Severity

### Current Modalities

The assessment of mitral regurgitation severity relies on a multiparametric approach in which several qualitative and quantitative parameters are taken into consideration (i.e., valve morphology, color Doppler, PW and CW Doppler, pulmonary venous flow, left ventricular and atrial volumes etc.). Current cut-offs for intervention remain based on 2D TTE and TEE [25].

The most frequently used quantitative parameter is proximal isovelocity surface area (PISA). From PISA, the effective regurgitant orifice area (EROA) and regurgitant volume (RVol) can be derived [25]. Two assumptions affect the accuracy of this method: the first is that the PISA method requires a circular geometry of the regurgitant orifice and a hemispheric shape of flow convergence. However, in most cases, the geometry of the regurgitant orifice is either slit-like, irregular, or composed of multiple defects of coaptation. The second assumption is that the amount of regurgitation remains constant throughout the entire systole so that the frame where the maximum hemispheric PISA is measured represents the entire regurgitant volume. Mitral regurgitant flow is dynamic with variable duration, convergence zone diameter, and conformational changes in the regurgitant orifice during the ejection period. To overcome this limitation, previous studies integrated changes in PISA with continuous-wave Doppler-acquired MR velocities. These attempts are manual and time-consuming, and the accuracy and reproducibility of these measurements are limited [26].

**Figure 14:**
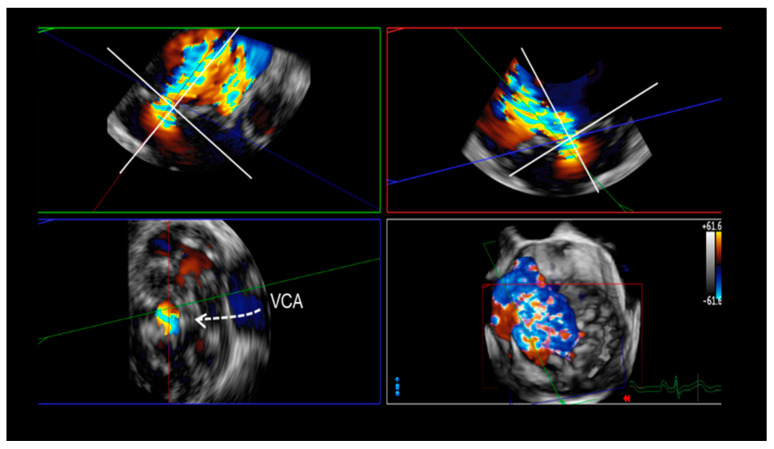
Two orthogonal cross₋sections are positioned parallel to the direction of the regurgitant jet; a third cross-sectional plane is perpendicularly oriented to the jet and moved along the jet direction until the cross-sectional area at the level of the maximal VCA is visualized. The frame with the largest VCA in systole is measured by direct planimetry.

Three-dimensional TEE measurement of the vena contracta area (3D VCA) seems to be the most trustworthy method for evaluating the degree of insufficiency. The method can be considered a surrogate of EROA, and it uses 2D TEE images derived from 3D volumetric data sets. Three-dimensional VCA seems to be independent of geometric assumptions (Figure 14). The accuracy of 3D VCA and the derived regurgitant volume have been validated against 2D volumetric Doppler methods and cardiac MRI. This method appears reliable in cases with eccentric MR jets as well as in cases with multiple regurgitant orifices. Importantly, the cut-off value of 0.41 cm^2^ yielded 97% sensitivity and 82% specificity in differentiating moderate from severe MR [27,28].

## 6. A New Tool

**Figure 15:**
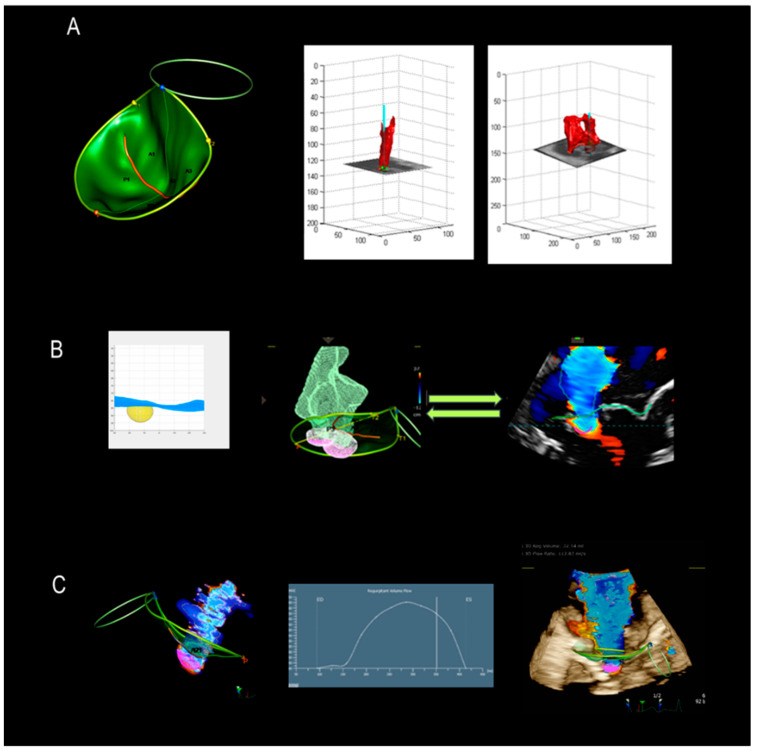
(**A**) The first step is the automated reconstruction of the volume rendering surface of the MV throughout the entire systole. The color flow is displayed in every frame and shape; the number and position of the orifice/s are assessed using the intersection of CF Doppler and MV surface rendering. (**B**) The second step is that the algorithm fills the orifice(s) with hundreds of tiny pinholes, and then generates a model of the convergence zone, displaying it as a purple mesh. This shape is compared to CF Doppler jet(s) in all systolic frames. The system can be reshaped by adding/removing pinholes to create a perfect match with 3D color Doppler. (**C**) The left panel shows Auto CFQ; in the middle panel, the graft shows the variation of regurgitant flow during systole; and the right panel shows the fusion of the 3D TEE image, surface rendering, and Auto CFQ.

A few months ago, a new system for quantifying MVR was launched and is currently available in clinical practice. This system benefits from the extensi ve use of AI. A description of the new methodology, called 3D Auto Color Flow Quantification (CFQ) (Philips Research Medisys, Suresnes, France), is beyond the scope of this review. In brief, the system can be divided into two steps: the first step is the automated reconstruction of the volume rendering surface of the MV throughout the entire systole. The color flow is displayed in every frame and shape; the number and position of the orifice/s are assessed using the intersection of CF Doppler and MV surface rendering area. In the second step, the algorithm fills the orifice(s) with hundreds of tiny pinholes, generating a model of the convergence zone, displayed as a purple mesh. This shape is compared to CF Doppler jet(s) in all systolic frames and reshaped by adding/removing pinholes to eventually create a perfect match with 3D color Doppler [29]. This new algorithm allows quantification in all types of MVR, including difficult cases with multiple jets and constrained or incomplete jets. However, future validations of this new modality need to involve larger numbers of patients and several institutions before it can be claimed that Auto CFQ is a real breakthrough in the assessment of MVR (Figure 15).

## 7. New Mini TEE Probe

**Figure 16:**
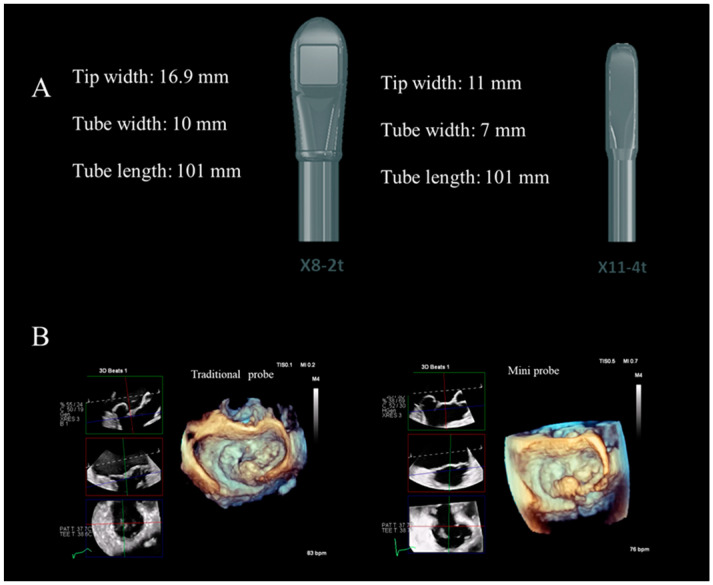
(**A**) Size comparison between traditional (left panel) and mini probes (right panel). (**B**) Comparison of image quality between the traditional probe (left panel) and mini probe (right panel) in the same case.

Both 2D and 3D TEE are semi-invasive imaging techniques. In the echo lab, the vast majority of TEE procedures are performed with moderate sedation through an intravenous line and topical anesthetic spray. Probe insertion is the critical and most invasive step. The TEE probe is inserted into the back of the pharynx through a bite block. A small percentage of patients (between 2–3% in our experience) do not tolerate probe insertion and require deeper sedation, usually with the support of anesthesiologists. A larger percentage of patients (5–10% in our experience) do not tolerate a long examination (>10 min). Over the years, attempts have been made to use small-sized pediatric probes in adult patients. While the introduction and tolerance of the mini probes have significantly improved, the image quality has worsened due to the lack of transducer contact with the esophageal wall and the limited number of crystals. A new Matrix Mini-Probe X 11-4T (Philips-Andover, Andover, MA, USA) is now available in clinical practice. A description of the engineering characteristics of this new mini transducer is beyond the scope of this review. In DMVR, this new mini transducer has shown an image quality equivalent to that of the traditional-sized transducer, optimal tolerance, and constant contact of the transducer with the esophageal wall. The advantages of easy insertion and longer tolerability should allow the use of this mini probe during transcatheter edge-to-edge procedures avoiding intubation and general anesthesia (Figure 16).

If these qualities are confirmed in large populations and many institutions, this new tool will certainly become part of the vast armamentarium available in echocardiography.

## 8. Conclusions

In this review, we have summarized the key role of 3D TEE in assessing DMVR by describing various morphological scenarios that reflect everyday experiences in an echocardiographic laboratory. Moreover, we have evaluated the impact on DMVR assessment of new technologies (photorealistic and transparent displays, surface rendering parametric color maps, new algorithms for MVR quantification) and the new mini-TEE probe. The authors are well aware that 2D TEE will continue to be indispensable for assessing DMVR by the large quantity of extra-valvular data provided by this modality. However, at the same time, we firmly believe that acquiring 3D data sets must become a standard part of the DMVR examination due to the reliable and adjunctive information they provide on valve morphology.

## Figures and Tables

**Table 1:** Developments.

Development	Description
New generation of piezoelectric crystals	More uniform atomic structure with fewer impurities and boundaries Greater efficacy in converting electrical energy into mechanical energy Reduction in the production of heat Larger spectrum of frequencies Better flexibility between resolution and penetration
Miniaturization of piezoelectric crystals	Crystals as small as 200 microns 3000 active elements in a traditional-sized transthoracic transducer 2500 active crystals in a traditional-sized transesophageal transducer
Fully sampled matrix array transducer	Thousands of elements active simultaneously Micro-beamforming circuitries whereby a small group of elements (i.e., patches of 25 crystals) combine their output to a single connecting wire 128 wires connecting 3000 crystals to the mainframe
Miniaturization of electronic circuitries	8 million electronic devices and thousands of microchannels inside a transthoracic and transesophageal probe Active cooling system minimizing heat generation without reducing transmit power

**Table 2:** Phenotypes of degenerative mitral valve regurgitation.

Phenotype	Description	Image
Fibroelastic deficiency	This phenotype is characterized by a deficient production of collagen, elastin, and proteoglycans. Leaflets preserve their own three-layer arrangement, but upon surgical inspection, they appear fragile and translucent. The etiology of connective tissue deficiency in FED is unknown, but it has been suggested that it may be the result of an accelerated ageing process. The cause of regurgitation is the rupture of one or more primary chordae tendineae, which usually involves a single scallop. The figure shows a small P2 prolapse in an otherwise apparently normal MV.	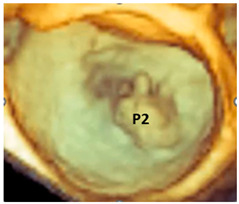
Fibroelastic deficiency plus	This phenotype is characterized by a single scallop prolapse, but the involved scallop is redundant and affected by myxomatous degeneration. The remaining scallops are normal. Qualitative histological lesions in the prolapsed tissue of FED plus are similar to those of Barlow’s disease. It has been suggested that FED plus may be considered a sort of worsening stage of FED, leading to the hypothesis that the myxomatous changes could also be secondary to jet lesions. The figure shows a large P2 prolapse with ruptured chordae tendineae (arrows).	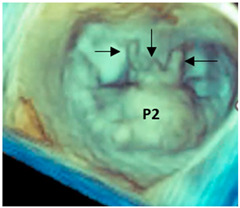
Forme fruste	In this phenotype, the entire posterior leaflet is redundant and is affected by myxomatous degeneration, while the anterior leaflet is macroscopically and histologically normal. It can be speculated that this phenotype is an incomplete form of Barlow’s disease. The figure shows multiple prolapses (asterisks) of the posterior leaflet.	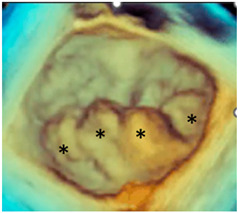
Barlow’s disease	Barlow’s disease is the result of an abnormal accumulation of myxomatous substances (mainly proteoglycans) in the spongiosa layer, and a simultaneous disruption of collagen fibers in the fibrosa layer. As a consequence, the three-layered arrangement of the leaflets is lost. The characteristic macroscopic appearance, either in pathological specimens or in the operating room, is that of a thick, bulky, redundant leaflet, elongated chordae tendineae, and annular dilatation. The excess leaflet tissue leads to the displacement of both leaflets beyond the annulus, with a lack of coaptation and consequent MR. The figure shows an example of Barlow’s disease seen from a tangential view largely protruding into the left atrium. LAA = left atrial appendage, AML = anterior mitral leaflet; PML = posterior mitral leaflet.	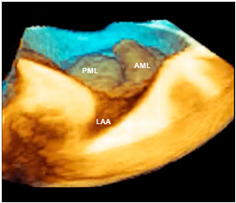

**Table 3:** Advantages of 3D TEE.

Advantages of 3D TEE	Description	Image
Long segment of catheter	The pyramidal data set embraces large volumes of the 3D space and allows the visualization of long segments of guide catheter (GC) without moving the probe. Moreover, small details such as the double ring at the tip of the GC can be visualized. MCDS = mitral clip delivery system.	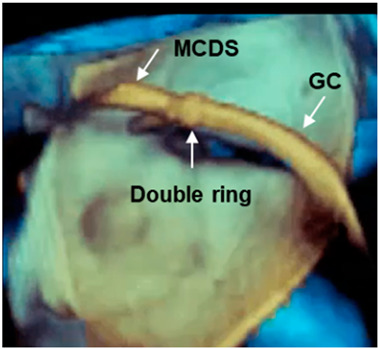
Advancement and steering of device in the left atrium	3D TEE allows the visualization of real-time, three-dimensional images of the motion of the GC and clip delivery system into the left atrium towards the MV. The dotted arrows in panels A–C point at the motion of the catheter. The arrows in panel D point at the arms of the clip. Of note is that the spatial relationship between the device and MV is easily understandable.	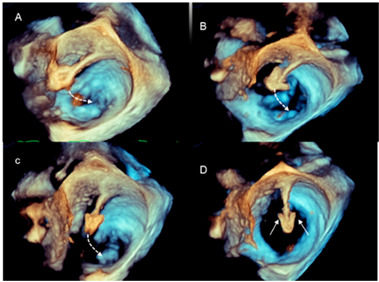
Positioning the arms perpendicular to the coaptation line	Before the advent of 3D TEE, this step was guided by 2D TEE in a transgastric short-axis view at the level of the tip of the mitral leaflets. This cross-section is often difficult to obtain. Conversely, 3D TEE provides images easily and they are immediately understandable by interventionists, who can manipulate the system while adjusting the position. Figure A–D show a still frame of the position of the clip delivery system.	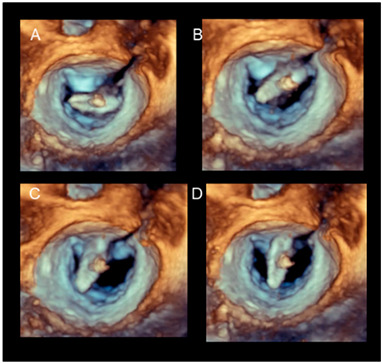
Panoramic view	3D TEE has the ability to include in a single panoramic view the mitral clip delivery system (clip, catheter) and the anatomic structures involved in the procedure with an acceptable volume rate.	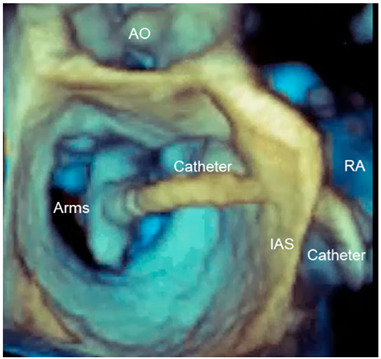

## Abbreviations and Acronyms

2D TTE	two-dimensional transthoracic echocardiography
2D TEE	two-dimensional transesophageal echocardiography
3D VCA	vena contracta area
AML	anterior mitral leaflet
CFQ	color flow quantification
DMVR	degenerative mitral valve regurgitation
EROA	effective regurgitant orifice area
FED	fibroelastic deficiency
MA	mitral annulus
MV	mitral valve
PML	posterior mitral leaflet
PISA	proximal isovelocity surface area
RVol	regurgitant volume
TEER	transcatheter edge-to-edge repair
